# Unraveling middle childhood attachment-related behavior sequences using a micro-coding approach

**DOI:** 10.1371/journal.pone.0224372

**Published:** 2019-10-29

**Authors:** Nadja Bodner, Guy Bosmans, Jasmien Sannen, Martine Verhees, Eva Ceulemans

**Affiliations:** 1 Quantitative Psychology and Individual Differences Research Group, Faculty of Psychology and Educational Sciences, KU Leuven, Leuven, Belgium; 2 Clinical Psychology Research Group, Faculty of Psychology and Educational Sciences, KU Leuven, Leuven, Belgium; 3 Parenting and Special Education Research Unit, Faculty of Psychology and Educational Sciences, KU Leuven, Leuven, Belgium; 4 Clinical Child and Family Studies, Vrije Universiteit Amsterdam, Amsterdam, The Netherlands; University of Portsmouth, UNITED KINGDOM

## Abstract

Attachment theory states that children learn to trust in their parent’s availability and support if they repeatedly experience that their parents respond sensitively to their needs during distress. Attachment is thus developed and shaped by day-to-day interactions, while at the same time, each interaction is a momentary expression of the attachment relation. How attachment-related behaviors of mother and child follow upon each other during interactions in middle childhood, and how these sequences differ in function of attachment quality, has hardly been studied up to now. To fill this gap, we analyzed the micro-coded interaction of 55 mother-child dyads (27 girls, 28 boys, mean age: 10.3) after a standardized stress-induction. Results reveal that all mother-child dyads show a loop between positive mother and child behaviors. This pattern is complemented with a loop of negative mother and child behaviors in low-trust and more avoidantly attached children: these children tend to handle negative mother behavior less well as they show more negative behavior and less positive behavior in response to negative maternal behavior. More anxiously attached children also show less positive behavior, but react positively on collaborative interactions. The micro-coded interactions thus reveal important insights that inform practitioners and advance attachment theory.

## Introduction

According to attachment theory, children learn to trust in their mother’s availability and support if they repeatedly experience that she responds sensitively during distress [1]. Children that, in contrast, experience their mother as unpredictable, unavailable or harsh, are more likely to become insecurely attached. Throughout development, attachment is continuously shaped by the daily interactions of mother and child. At the same time, their behaviors in stressful situations are momentary expressions of their attachment relation [e.g., 2], where children may for instance seek support (secure attachment), overly rely on themselves (avoidant attachment) or launch an exaggerated call for help (anxious attachment). Attachment researchers often assess infants’ attachment development by coding infant behavior in stressful mother-child interactions [3,4], because this approach yields valid and interesting results, but also because this is the only feasible measurement strategy in this age group.

Behaviors become more diverse, subtle and complex with increasing age, which makes it harder to observe and identify attachment-related behavior [5,6]. Also, the behaviors of mother and child get more intertwined and reciprocally dependent [6,7], implying that both the singular behaviors of mother and child and the sequencing of how mother and child react on each other should be combined to assess attachment quality [8]. The scarce observational studies on attachment in middle childhood (and adolescence) use macro-coding approaches in which certain aspects of the behavior of mother and/or child during an interaction are rated. The rating scales measure attachment-relevant dimensions, such as the amount of parental sensitivity and child responsiveness (Emotional Availability Scale) [9], security, avoidance, ambivalence and disorganization of the child (Iowa Attachment Behavior Coding) [10], parent’s or adolescent’s punitive or role-confusing behavior (Goal‐Corrected Partnership in Adolescence Coding System) [11,12], secure, ambivalent and disorganized attachment behavior of the child (Middle Childhood Attachment Strategies) [5]. Clearly, such macro-coding approaches provide detailed and rich scores. However, they only indirectly permit to account for sequencing since the full interaction is summarized in one score per rating scale. To study the temporal sequencing of behavior in interactions, other subfields of developmental research have adopted sequential coding schemes and analysis approaches [13], in which the behavior is coded at multiple time points during the interaction (time-based coding) or every time a change occurs (event-based coding), allowing to investigate how behaviors elicit one another. A good example of event-based coding is the study of Dishion, Spacklen, Andrews, & Patterson [14], who inspects how peers react to each other’s rule-breaking messages during an interaction by coding the reaction to each message separately. The study of Van keer et al. [15] in which the behavior of young children with a significant cognitive and motor developmental delay and their parents are coded every second, provides an example of time-based coding. With the current study we aim to combine the best of both worlds: Like the sequential coding and analysis approaches, we investigate within interaction changes in behavioral reactions of mother and child. In line with the macro-coding based attachment studies, we use detailed and rich coding schemes that measure a variety of attachment-relevant behaviors.

### Attachment in middle childhood

During middle childhood, the attachment relation between children and their parents changes due to neural and cognitive development (associated with adrenarche) [16]. Parents and children enter into a ‘supervision partnership’ in which problems are solved together in a collaborative alliance, and decisions are negotiated [17]. These changes alter (1) which situations elicit attachment-related behaviors, (2) which behaviors can be considered attachment-related, and call (3) for studying besides the frequencies of specific behaviors also the likelihood of their sequencing.

#### Situations that elicit attachment-related behavior

Attachment behavior can be observed in stressful situations in which the attachment figure is present [1,3,18,19]. The degree to which children turn towards their parents in such situations provides useful information on how securely they are attached. Thus, to observe attachment-related behavior, a certain amount of stress is necessary. The factors that render a situation stressful, however, change in middle childhood. While attachment research in infancy builds upon typical infant threats (e.g., “strange people, strange places, … long duration of separation or larger distance from the attachment figure”, p.12) [6], simple separation and reunion procedures become less efficient in eliciting the urgent need to reassure parents’ availability in middle childhood [5,20]. To initiate support-seeking behavior, the separation needs to be complemented with other stressors, for example, with developmental task-relevant distressing themes, such as academic failure, not being able to measure up with expectancies, social conflicts or being rejected by peers [6,18,21].

In the current study, we relied on the ‘comparison with peers’ and the ‘inability to measure up with expectancies’ themes to elicit distress, by asking children to solve an unsolvable puzzle, while telling them that it had been solved easily by other children [22]. To maximize distress, the children worked alone, separated from their mothers. After five minutes mother briefly entered the room and handed a bell to the child, with which the child could call her to help. Through this manipulation, we aimed to transform the unspecified stress reactions into meaningful attachment-related behavior (i.e., proximity seeking). Then, mothers and children were reunited, and worked together on the task for three additional minutes. This interaction between mother and child was videotaped.

#### Attachment-related behaviors

In middle childhood, children still display the same attachment behaviors as younger children and thus often seek physical contact when they are distressed to be comforted by the mother [18,23]. However, attachment-related behavior expands beyond easy detectable approaching child behavior and comforting mother behavior. For instance, mother and child can also assure support by simply exchanging gazes [6]. A wide range of more subtle behaviors can thus be considered support-seeking or caregiving, depending on the behavior sequence in which they occur.

To fully account for this diversity, this study proposes the Middle Childhood Attachment Micro-observation system (MCAM) to code ‘positive child behavior ‘ (encompassing engagement, positive affect, involving mother in a positive way), ‘positive mother behavior’ (including attention, responsivity, positive affective communication, structuring in a positive or task related way), ‘negative child behavior’ (involving the mother in a negative way, controlling/contact-maintaining, avoidant, and resistant behavior), and ‘negative mother behavior’ (structuring in a directive way, covert or overt hostility, non-contingent reaction). Additionally, the coding system contains three task (i.e., puzzle) related behaviors: ‘child works alone’, ‘mother works alone’, ‘mother and child work together’. All these behaviors are coded in two-second intervals, yielding intensive longitudinal data that enable us to trace the relative frequencies of the behaviors as well as their temporal sequencing [24].

#### Tracing behavior frequencies and sequences

Based on attachment theory, the frequencies and temporal sequencing of behaviors in stressful mother-child interactions can be expected to be related to attachment quality. Building on the mutual responsive orientation concept in early childhood [25], we conjecture that positive expressions of securely attached children and their mothers follow upon each other in an attuned way in that responsive mothers for example support their children who, in return, positively involve their mothers while working on the task. Negative feelings can safely be expressed, but will easily be resolved [2], as securely attached children rely on their parents to regulate their emotions [26]. In contrast, children scoring high on attachment anxiety, will probably exhibit more negative behavior that also perseveres longer, using the negative behavior to elicit proximity [27]. Whereas we expect mothers of the more anxiously attached children to react in an unpredictable way to calls for help, we conjecture that mothers of children scoring high on avoidant attachment, in contrast, will not engage or respond in a more negative way (insensitive, withdrawn or even hostile). Avoidant children are expected to interact less, as they do not quickly seek proximity or support in times of distress [27], ignore negative mother behavior and mask their distress by displaying object oriented behavior [28].

The aim of this paper is to test these hypotheses by adopting a micro-coding approach and by (1) quantifying how frequently the behaviors of the MCAM occur and how likely they follow upon each other in a sequence and (2) relating these frequencies and sequencing likelihoods to self-reported attachment. To capture the sequencing likelihoods, we adopted the approach from Bodner et al. [29] and calculated Jaccard similarity indices [30,31] between one behavior at time point *t* and another behavior at time point *t*+1. Comparing these patterns to self-reported attachment we aim to shed light on whether and how individual differences in self-reported attachment are reflected in interaction patterns between mothers and their children during a distressing task.

## Methods

### Participants

We analyzed interaction data from a study conducted by Dujardin et al. [22]. Out of the 98 mother-child dyads for which all data were correctly collected, we selected 55 dyads for which the recording quality of the videotaped mother-child interaction was sufficient and that represented all observed scores on the PIML questionnaire (see below), but overrepresenting the higher and lower ones. The 55 selected dyads (27 girls, 28 boys) did not differ significantly from the 43 not selected dyads with respect to child age and gender, maternal marital status (87.3% married or living together), or educational level (83.6% bachelor/ master degree). The children were aged between eight and 12 (mean age: 10.3 years, *sd*: 0.93). In 54 dyads the mother was the biological mother, one child was adopted.

### Material

#### Middle Childhood Attachment Micro-Observation System (MCAM)

To code the positive and negative attachment-related behaviors that mother and child displayed in each two-second interval of the interaction, we developed the Middle Childhood Attachment Micro-Observation System (MCAM). This system captures proximity-seeking behaviors as well as cooperative problem-solving behaviors. The overarching positive and negative behavior categories are therefore divided further into subcategories inspired by the Emotional Availability Scales, middle childhood version (EAS) [9,32] and the strange situation procedure [3]. As both coding systems were originally developed for macro-coding interactions (i.e., one overall score of the complete interaction), they had to be adapted by including detailed descriptions that help to detect the behavior in small intervals. Table 1 provides an overview of the (sub)categories we coded and the original scales by which they were inspired. The detailed coding schema is available on request. During coding, we registered for each interval the presence ‘1’ or absence ‘0’ of the subcategories, allowing for the co-occurrence of behavior from several subcategories and even from different overarching categories (i.e., per interval more than one behavior (sub)category can be registered). Afterwards, the binary scores on the four main categories (first column of Table 1) were obtained by checking whether at least one of the subcategories of the main category was coded as present. Next to the positive and negative behaviors of mother and child, we also coded who was working on the puzzle. Summarizing, each dyad was scored for every two-seconds interval with respect to seven categories: ‘positive mother behavior’ (M+), ‘negative mother behavior’ (M-), ‘positive child behavior’ (C+), ‘negative child behavior’ (C-), ‘child works alone’ (CAlone), ‘mother works alone’ (MAlone), and ‘mother and child work together’ (Together).

**Table 1:** Main categories and subcategories of the MCAM, also indicating by which subscales of the emotional availability scale (EAS) [9] or strange situations procedure [3] they are inspired.

Main Category (as used in analysis)	Subcategory (as used for coding)	Scale
Positive mother behavior (*M+*)	Attention or behavior directed towards the child	Sensitivity (EAS)
	Responsivity: Adequate responsiveness to the social and emotional expressions of the child.	Sensitivity (EAS)
	Positive affective communication	Sensitivity (EAS)
	Mother provides structure in a positive or neutral way	Structuring^1^ (EAS)
	Task related structuring	Structuring^1^ (EAS)
Negative mother behavior(*M-*)	Structuring in a directive, negative way	Structuring^1^ (EAS)
	Covert hostility	Hostility (EAS)
	Open hostility	Hostility (EAS)
	Non-contingent reaction	--
Positive child behavior (*C+*)	Child engages in the relationship with mother	Responsivity (EAS)
	Child shows positive affect	Responsivity (EAS)
	Child intends to involve the mother in a positive or neutral way	Involvement^1^ (EAS)
Negative child behavior (C-)	Child involves mother, but content is negative	Involvement^1^ (EAS)
	Controlling behavior intended to maintain contact	Controlling contact maintain (Strange Situations)
	Proximity and interaction avoidant behavior	Avoidant (Strange Situations)
	Proximity and interaction resistant behavior	Resistant (Strange Situations)
Mother works alone (MAlone)	Mother works on the puzzle alone (MAlone)	--
Child works alone (CAlone)	Child works on the puzzle alone (CAlone)	--
Mother and child work together (Together)	Child and mother work on puzzle together (Together)	--

^1^ As structuring can involve positive or negative behavior, we include both positive and negative structuring. The same reasoning holds for involvement.

The videos were coded by two trained raters (33 videos by the first rater, 22 by the second) who were blind for all other dyad information. Coding a 3 min video in 2 sec-intervals takes trained raters about 1.5 hours. To investigate inter-rater agreement, two videos, together consisting of 169 intervals, were coded independently by both raters. Cohen’s Kappa [33] was calculated across all seven categories (M+, M-, etc.). The kappa score amounts to .871 (*p* < .001;CI_95_ = [.836;.906]) suggesting “almost perfect agreement” [34]. Next, following a recommendation of Bakeman, McArthur, Querda & Robinson [35], we compared the focal measures of the study, the values of the relative frequencies and the likelihood of the behavioral sequences. We correlated these measures (calculated for each dyad separately) of the two coders, obtaining a value of .99 (*p* < .001;CI_95_ = [.974;.995]) for the relative frequencies and of 0.95 (*p* < .001;CI_95_ = [.917;.965]) for the likelihood of the sequences.

#### Attachment questionnaires

Confidence in maternal care was measured with the People In My Life Questionnaire [PIML; 36], using the Dutch translation by Bosmans, Braet, Koster, & De Raedt [37]. The trust subscale contains ten statements like “I can count on my mother to help me when I have problems” which have to be rated on a 4-point Likert scale, ranging from 1 (almost never true) to 4 (almost always true). The PIML is a reliable and valid measure for primary school children [36]. Cronbach’s *α* for the trust subcale is .80 in our sample.

One child scored on average very low on trust (1.5) in comparison to the others (*z*-score: -6.2). The remaining 54 children were fairly homogeneous (see Table 2). To not distort the analyses, we excluded the low-trust mother-child dyad from the sample analyses. In the supporting information, we report its relative frequencies and sequences separately as they may provide valuable insight into low-trust dynamics.

**Table 2:** Mean, standard deviation (SD), range, and (partial) spearman correlations of the self-reported attachment scores.

	Trust	Avoidant	Anxious
Mean (SD)	3.51 (0.32)	2.44 (0.84)	2.11 (0.79)
Range	[2.8 ; 4 ]	[1 ; 4.5]	[1 ; 3.9]
Avoidant	-.51*** (-.39**)		
Anxious	-.38** (-.13)	.56***	

Note: *p* < .05

**p < .01.

***p < .001. Spearman correlations are used for all bivariate and partial correlations as the scores of trust and anxiety are not normally distributed (shapiro-test *p*< < .001)

Attachment anxiety and attachment avoidance in the mother-child relationship were measured with the Experience In Close Relationship–Revisted version for children (ECR-RC). We used the Dutch translation [38]. The anxiety subscale consists of 18 items and assesses the preoccupation with proximity and the fear to be abandoned or rejected (e.g., ‘I worry about being abandoned by my mother’). The 18 items of the avoidance subscale measure self-reliance, discomfort with closeness and avoidance of intimacy (e.g., ‘I prefer not to tell my mother how I feel deep down’). All items are rated on a Likert-scale from 1 (not at all) to 7 (very much).The ECR-RC has a high level of internal consistency and correlates with depressive symptoms, emotion regulation strategies [39] and parenting dimensions [40]. Cronbrach’s α in this study are .86 for anxiety and .85 for avoidance. Table 2 shows that the mean scores of the 54 children (excluding the low-trust dyad) across the 18 items are fairly low for both subscales. The avoidant and anxious subscales are positively correlated, while trust correlates negatively with both of them (Spearman correlations are used for all bivariate and partial correlations as the scores of trust and anxiety are not normally distributed; shapiro test *p*< < .001). For the anxious subscale, this correlation becomes non-significant, when controlled for avoidance.

### Procedure

**Fig 1:**
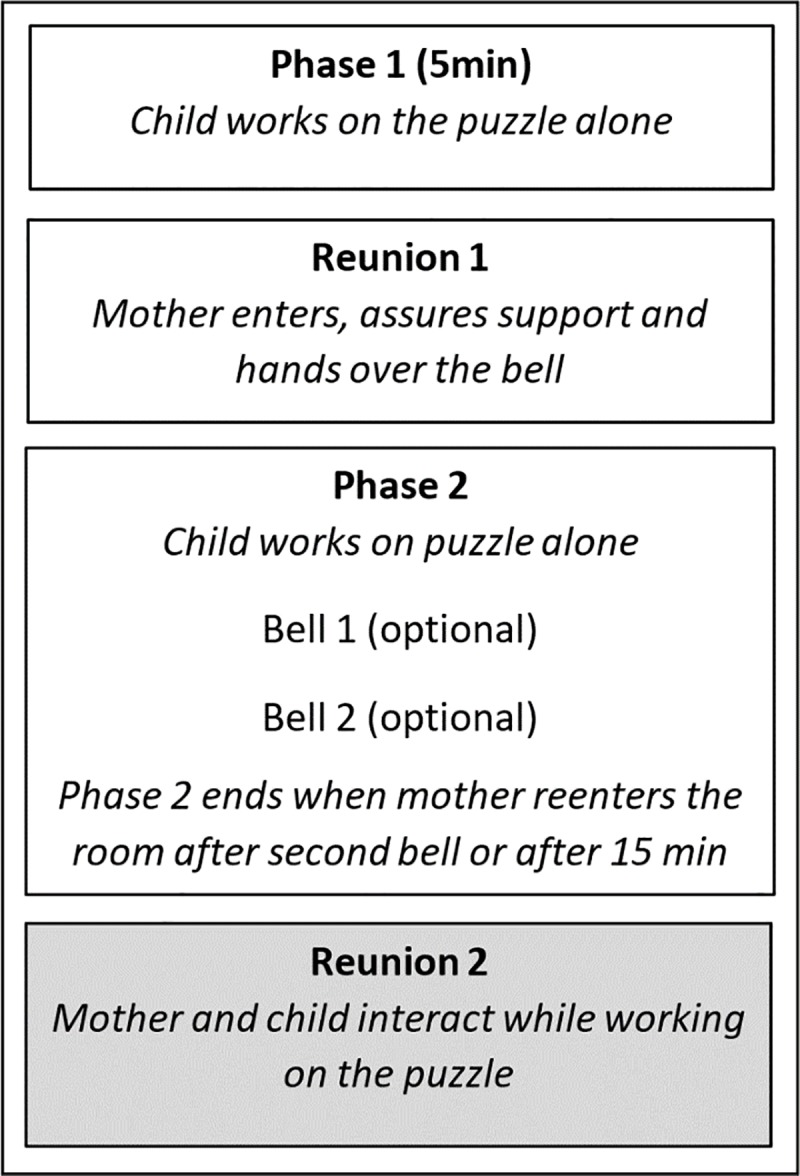
Rush hour task procedure.

The mother and child dyads were invited to a lab session, of which full details can be found in Dujardin et al. [22]. Mother and child were informed separately about the goal and the procedure of the study. After the mother had signed the informed consent form, the child completed the ECR-RC and the PIML. Next, they turned to the stress eliciting task based on the Rush Hour Traffic Jam Puzzle^®^. An unsolvable version of this logic puzzle was developed by [22]. To elicit the need of maternal proximity, the child first worked on the puzzle alone, but could call the mother with a bell (handed over during the first reunion, see Fig 1). After the child called the mother for the second time or after 15 minutes, mother entered the room and worked together with her distressed child on the task. Although the mother knew that the puzzle was unsolvable, she was asked not to tell the child and to support the child. The study ‘Influences of stress-reaction on attachment in primary school children‘ (S53162) has been approved by the Medical Ethics Committee UZ KU Leuven on 8/04/2011 (ML7312).

The procedure induces distress as evidenced by a significant increase of skin conductance level, and elicits support and proximity-seeking behavior, since most children pressed the bell to call their mother [22]. We assumed that the attachment-related behavior is best observable if the stress is totally induced (i.e., towards the end of the task). For this reason, we focused on the behaviors of mother and child during the second reunion, which lasted for three minutes.

### Analysis

To unravel the interaction patterns between mother and child, we quantified the relative frequency (how often do the seven behavior categories occur) and the temporal sequencing of these behaviors (how likely is it that they follow upon each other) for each dyad separately. The relative frequency of the behavioral categories is operationalized as the proportion of time intervals in which they are shown.

To shed light on the likelihood of temporal sequencing, we calculated a normalized Jaccard similarity index [41]:

$$Jac_{Norm}=\frac{Jac_{Obs}-Jac_{Exp}}{1-Jac_{Exp}}$$ (1)

Jac_Obs_ is the observed Jaccard index [30,31], reflecting the association between a first behavior and a second behavior in the next time interval:

$$Jac_{Obs}=\frac{n_{11}}{n_{11}+n_{10}+n_{01}}$$ (2)

where n_11_ denotes how many times the first behavior is followed by the second behavior in the next interval, n_10_ how many times the first behavior is expressed, but not followed by the second behavior, and n_10_ how many times the second behavior is shown, without being preceded by the first behavior. Jac_Exp_ expresses the Jaccard value that we would expect if no systematic association was present, taking the relative frequencies of both behaviors, denoted by p_1_ and p_2_, into account:

$$Jac_{Exp}=\frac{p_{1}*p_{2}}{1-(1-p_{1})*(1-p_{2})}$$ (3)

Like Kappa, a Jac_Norm_ value of 0 implies that this behavioral sequence occurs no more than expected by chance, whereas a value of 1 indicates that the two behaviors always follow each other.

All calculations and plots have been executed in R Version 3.6.1 (free software available on https://cran.r-project.org/) using R-studio Version 1.2.1335 (https://www.rstudio.com/). The R-script and the dataset is openly available on OSF (https://osf.io/59xkf/ *)*.

## Results

First, we examined the distribution of the relative frequencies and sequencing likelihoods in the main sample of 54 children (excluding the low-trust dyad; see Methods), to gain insight into which behaviors and sequences typically occur. Next, we related the differences in these measures to self-reported attachment. How the measures of the low-trust dyad differed from the rest of the sample can be consulted in the supporting information.

### Typical behaviors and sequences

**Fig 2:**
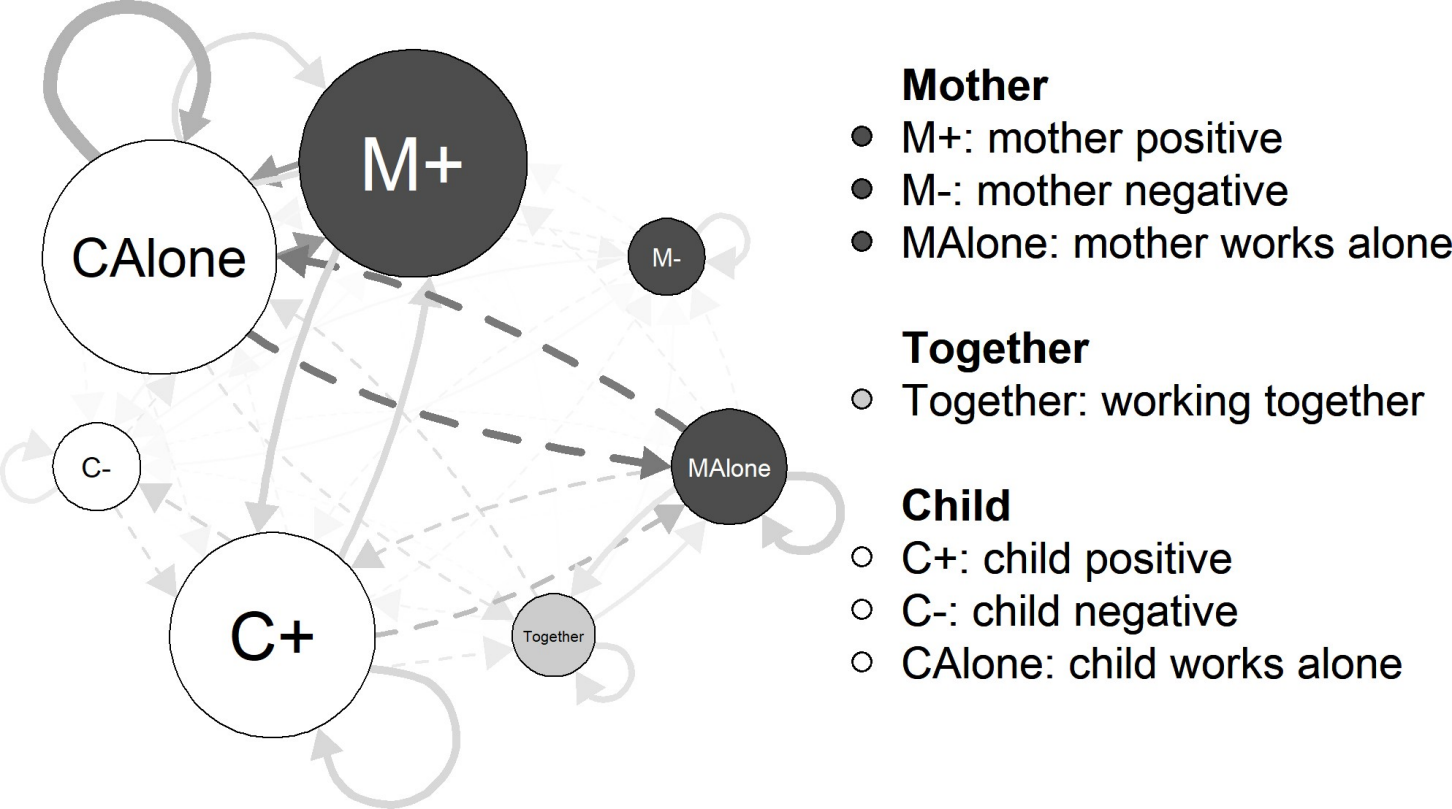
Average network of the main sample. Categories are depicted as nodes, sequencing likelihood as arrows between them. Node size is adapted to the average relative frequency of the category. Please note that a minimum node size was introduced, to warrant the readability of the node labels. Thickness and saturation of the links depict degree of deviance from random behavior. Dashed lines indicate behavior that is shown less than expected. Auto-loops are downscaled to depict the sequencing likelihoods between different behavior categories more clearly.

Table 3 shows the relative frequencies of the seven behavior categories. For each pair of these categories, Table 4 presents the number and proportion of dyads in which the sequence is shown more than expected by chance, the average likelihood of the sequence, and its standard deviation and range. To facilitate interpretation, Fig 2 visualizes the average relative frequencies and sequence likelihoods in a network figure, following [29]. This figure depicts the average relative frequency of the seven behavior categories (size of the nodes) and sequencing likelihoods (thickness and saturation of the directed links between the nodes, with solid/dashed links indicating that a sequence occurs more/less often than expected). M+ and C+, and CAlone were observed most frequently, while negative behavior was coded less often. Regarding sequencing, we see that the C+→M+ and M+→C+ sequences are more likely than expected by chance. On the other hand, switches between the puzzle behavior of child (CAlone) and mother (MAlone) occurred less often than expected by chance.

**Table 3:** Relative frequencies of mother and child behaviors of the MCAM. Total time of occurrence, number of dyads showing the behavior (Dyads), mean, standard deviation (SD) and range (Min, Max) for the main.

		Total	Dyads	Mean	SD	Min	Max
**M+**		**2077**	**54**	**.45**	**.14**	**.13**	**.83**
	Attention	776	54	.17	.10	.02	.46
	Positive affective communication	311	48	.07	.07	.00	.27
	Responsivity	341	50	.07	.06	.00	.26
	Structuring (Pos./neutral)	129	36	.03	.04	.00	.19
	Task related structuring	798	51	.18	.10	.00	.42
**M-**		**95**	**18**	**.02**	**.06**	**.00**	**.38**
	Not-contingent reaction	54	13	.01	.04	.00	.29
	Structuring (directive/neg.)	21	5	.00	.02	.00	.08
	Covert hostility	18	7	.00	.01	.00	.09
	Overt hostility	3	1	.00	.00	.00	.03
**MAlone**		**602**	**47**	**.13**	**.11**	**.00**	**.36**
	Mother alone	602	47	.13	.11	.00	.36
**Together**		**162**	**39**	**.04**	**.05**	**.00**	**.31**
	Together	162	39	.04	.05	.00	.31
**C+**		**1783**	**54**	**.39**	**.17**	**.06**	**.78**
	Engagement	725	54	.16	.08	.03	.35
	Positive affect	188	33	.04	.06	.00	.24
	Involving mother (pos./ neutral)	1079	54	.24	.14	.01	.56
**C-**		**244**	**38**	**.05**	**.08**	**.00**	**.39**
	Involving mother (neg.)	142	32	.03	.06	.00	.28
	Controlling/maintaining	0	0	.00	.00	.00	.00
	Avoidant	73	14	.02	.03	.00	.13
	Resistant	32	6	.01	.02	.00	.13
**CAlone**		**2179**	**53**	**.47**	**.22**	**.00**	**.92**
	Child alone	2179	53	.47	.22	.00	.92

**Table 4:** Sequences of mother and child behaviors. Number of dyads showing the sequence (Dyads), proportion (PropDy>0) of families showing the sequence more than would be expected by chance, mean and significance level (sig), standard deviation (SD) and range (Min, Max) for the main sample.

	Population (*n* = 54)						
	Dyads	PropDy>0	Mean	sig	SD	Min	Max
M+ **= >** M+	54	.94	.19	***	.11	-.05	.53
C+ = > M+	54	.78	.07	***	.10	-.17	.29
M- = > M-	18	.61	.15	**	.18	-.02	.65
MAlone = > MAlone	47	.83	.27	***	.20	-.04	.81
MAlone = > Together	36	.50	.04	*	.15	-.05	1
Together = > Together	39	.56	.17	***	.18	-.03	.66
M+ **= >** C+	54	.78	.08	***	.10	-.09	.31
C+ **= >** C+	54	.98	.24	***	.12	.00	.51
M+ **= >** C-	35	.51	.01	*	.06	-.05	.30
C- **= >** C-	34	.59	.12	***	.15	-.03	.48
CAlone **= >** CAlone	53	.98	.47	***	.18	-.02	.81

*Note*: an upper-tailed one sample t-test was used.

*p<0.05

**p<0.01

***p<0.001, only links with *p*<0.05 are reported, full table is available in supporting information (S2 Table).

### Correlating the relative frequencies and sequencing likelihoods with self-reported attachment

Most of the relative frequencies (Table 3) and sequencing likelihoods (Table 4) differed considerably across the dyads. To investigate how these differences relate to attachment, we correlated them to trust (PIML), attachment avoidance and anxiety (ECR-RC). The resulting correlations can be consulted in Table 5 (relative frequencies) and Table 6 (sequencing likelihood).

**Table 5:** Correlations between relative frequency of behavior and self-reported attachment (for Avoidance and Anxiety partial correlations–correcting for each other—are provided in brackets).

	Trust	Avoidance	Anxiety
M+	.04	-.01 (.07)	-.12 (-.14)
M-	.05	.12 (.09)	.08 (.02)
Malone	-.09	-.02 (-.11)	.13 (.16)
Together	.11	.12 (.13)	.02 (-.05)
C+	.17	-.39** (-.2)	-.42** (-.27)
C-	-.05	.16 (.12)	.12 (.03)
CAlone	.24	-.01 (-.02)	.01 (.02)

*Note*: significance level:

**: *p*<0.01.

**Table 6:** Spearman correlations for Trust, Avoidance and Anxiety and partial correlations for Avoidance and Anxiety (controlling pairwise for each other) between sequences of behavior and self-reported attachment (*p*-values between brackets).

	Trust	Avoidance	Avoidance (partial)	Anxiety	Anxiety (partial)
M+ = > M+	-.36**(.01)	.36**(.01)	.42**(0)	.03(.86)	-.23(.09)
M+ = > M-	.3*(.03)	-.21(.12)	-.16(.24)	-.14(.31)	-.02(.86)
MAlone = > M-	.15(.29)	-.35*(.01)	-.25(.08)	-.27(.06)	-.1(.51)
C- = > M-	-.5**(0)	.14(.4)	.08(.62)	.12(.45)	.05(.74)
MAlone = > MAlone	-.37*(.01)	.19(.2)	.14(.34)	.12(.41)	.02(.91)
M+ = > Together	-.19(.17)	.27*(.05)	.13(.34)	.3*(.03)	.18(.2)
C- = > Together	.02(.86)	-.17(.23)	-.02(.9)	-.28*(.05)	-.23(.11)
M- = > C+	.23(.1)	-.36**(.01)	-.31*(.02)	-.19(.17)	.01(.92)
Together = > C+	-.15(.27)	.12(.39)	-.07(.63)	.31*(.02)	.29*(.03)
M- = > C-	-.36*(.03)	.32*(.05)	.15(.36)	.37*(.02)	.24(.15)
Together = > C-	-.01(.92)	-.22(.12)	-.03(.85)	-.38**(.01)	-.31*(.03)
C- = > C-	-.39*(.01)	.2(.23)	.01(.97)	.35*(.03)	.3(.08)

*Note*: significance levels:

*: *p*<0.05

** p<0.01, *** p<0.001. All correlations are spearman correlations. Partial correlations with anxiety control for avoidance and vice versa. Please note that we only report links here where at least one correlation with a self-reported attachment was significant, for the other links please consult the supporting information (S3 Table).

### Trust

The relative frequency of the seven behavior categories was not related to trust. Regarding sequences, the auto-loops of M+, MAlone and C- correlated negatively with trust, indicating that in lower-trust dyads these behavior categories lingered longer than in higher-trust dyads. Similarly, the likelihoods of the M-→C- and C-→M- sequences were negatively correlated to trust. Both sequences were shown more often in lower-trust dyads. In higher trust dyads, in contrast, it was more likely that mothers switched from positive to negative behavior (M+→M-). (Visualization of the Trust network in S1 Text, S1 Fig)

### Avoidant attachment

The relative frequency of C+ was negatively correlated to avoidance: high-avoidant children showed positive behavior less often. The sequences M-→C-, M+→Together and the auto-loop of M+ were more likely in dyads with higher scores on avoidance, whereas Malone→M- and M-→C+ were less likely in these dyads. To account for the overlap of avoidance and anxiety (see Table 2), we also computed partial correlations, controlling for anxiety. Some of the above correlations were not significant anymore, suggesting that they occurred due to the shared variance of avoidance and anxiety. More avoidant dyads seem to be characterized by M-→C+ becoming less likely and by a stronger auto-loop of M+. (Visualization of the avoidant network in S1 Text, S2 Fig)

### Anxious attachment

The correlations show that C+ was shown less often in higher-anxiety dyads and C- lingered longer in these dyads. In higher-anxiety dyads, M-→C-, M+→Together and Together→C+ were more likely, whereas Together →C- and C-→Together were less likely. Only the different child reactions to Together appeared uniquely linked to children’s higher attachment anxiety after controlling for children’s attachment avoidance. (Visualization of the anxious network in S1 Text, S3 Fig)

## Discussion

This study aimed to investigate the relative frequencies and sequencing likelihood of mother and child behaviors in middle childhood and how those features relate to self-reported trust in maternal support, attachment avoidance, and attachment anxiety. To this end, we exposed the child to a stressful situation and coded the behaviors during the subsequent mother-child interaction with a micro-coding system, yielding binary scores on seven behavior categories.

### A positive behavior feedback loop in all dyads

An eye catching finding is that in all dyads (even in the separately analyzed low-trust dyad, see supporting information, S1 Table, S2 Table, S2 Text, S4 Fig) the positive child and mother behaviors follow upon each other more often than would be expected by chance. This finding is interesting because such positive interactions are mandatory for installing secure attachment. Indeed, sensitivity, responsiveness and positive affective communication—which are the main ingredients of the M+ category—should calm the child in distressing situations and create trust in maternal support [6]. Moreover, the high likelihood of M+→C+ and C+→M+ sequences could be interpreted as an expression of a mutually responsive orientation [25], implying close mutual interaction, which includes cooperative elements (e.g., ‘mother structuring task’, ‘child involving mother’) and positive affect. The concepts of mutually responsive orientation and secure attachment are closely related [42]. Whereas the inter-dyadic differences in the likelihood of these sequences are not related to scores on maternal Trust, Avoidance or Anxiety, children that score higher on attachment avoidance or anxiety showed less C+. One possible explanation is that these children might be more reserved towards their mothers [20], possibly in order to avoid disappointment as they do not expect their mothers to react responsively.

### Complementary negative behavior patterns in lower-trust dyads

Complementing this feedback loop of positive behaviors, a negative loop emerged in dyads lower in trust: The likelihoods of C-→M- and M-→C- were negatively correlated to trust. Moreover, the M-→C- sequencing strength was also positively correlated to attachment avoidance and anxiety. Based on theory, however, we could argue that these negative behavior pattern probably functions differently for more avoidantly and anxiously attached children. For more anxiously attached children, Cassidy [2] predicts M-→C- sequences, where these children express their anger with the mother to ensure their mother’s proximity. Cassidy’s view also predicts that these children are highly motivated to have positive interactions with their mother, which would fit with our findings in that Together→C+ sequences have a high and Together→C- sequences a low likelihood. More avoidantly attached children, in contrast, might use negative behavior to distance themselves from their attachment figure to protect themselves from new relational pain, see [2], for a similar line of reasoning. Another indication that avoidant children do not engage in reducing the distance can be seen in their lower M-→C+ sequencing likelihoods, which suggests that children are not trying to ameliorate the affective tone of the interaction with their mothers by showing positive behavior. Indeed, previous research has shown that securely attached adolescents display behaviors intended to safeguard the relationship [43], while avoidant adolescents get stressed by charged interactions and tend to avoid them [44]. This also links up with the results of Cassidy and Kobak [45] suggesting that less securely attached children tend to ignore the disturbing information implied in negative mother behavior and avoid asking for comforting, as they do not expect to receive it in the current attachment relation.

### Strengths and limitations

This study proposed a novel micro-coding approach and combined it with a standardized stress inducing situation to shed light on attachment-related interaction dynamics between mother and child in middle childhood. This approach has several strengths: First, the MCAM coding system builds upon the long–standing observational approach in infant attachment research [3,9]. Yet, it replaces the underlying macro-coding approach by a micro-coding approach. This micro-coding approach facilitates the quantification of the likelihood of behavioral sequencing and unveils insights into the more intertwined dyadic interaction dynamics that emerge in middle childhood [6]. Additionally, depicting these sequences and the relative frequencies in a network yields the unique opportunity to obtain a complete picture of the interaction and compare it between groups or across dyads [29]. Thus, the micro-coding approach is a valuable and promising new tool for attachment researchers and opens perspectives for further research.

As our study is the first to apply this micro-coding approach to attachment-related behaviors, some critical remarks need to be made. First, finding a positive feedback loop in all dyads (independent from their scores on self-reported attachment), seems a positive sign, especially, as we found this positive feedback loop also in the outlier (see supporting information). We can, however, not exclude, that this finding is due to our rather small community sample, in which all children have high trust values. Future research might apply the paradigm to a group of more insecurely attached children to check whether the findings replicate.

Second, the findings show distinct sequencing patterns for children high on trust, high on attachment anxiety and high on attachment avoidance. However, we should acknowledge that only a few correlations between the attachment measures and the patterns are significant and that the networks of anxious and avoidant attachment show considerable overlap. Again, our rather small community sample with overall high scores on trust and a considerable correlation between anxious and avoidant scores might explain the limited number of significant correlations when correcting for this overlap. The use of self-report questionnaires that imply a dimensional perspective on attachment may have artificially increased the impression that both insecure attachment styles correspond with partially overlapping behavioral sequences. Although some overlap has been noted when coding attachment behavior in a more categorical way [3], it might be useful for future research to also adopt a categorical measure like the Child Attachment Interview (CAI) [46]. Applying other measures of attachment would additionally allow to investigate how disorganized children interact with their mothers. The discussion on what disorganized attachment really refers to remains open [47] and this issue is even more problematic in middle childhood where research suggests that different disorganized attachment subgroups (like punitive-controlling, behaviorally disorganized, and controlling-caregiving children) might need to be distinguished [48]. In spite of the fact that the current study's approach might provide important leads to further identify how disorganized attachment is expressed in middle childhood, this seemed overly ambitious at this stage of the study. It would, however, be highly relevant to continue in this direction in future research.

Third, most of the child behaviors included under C- were observed in our study. However, the overall frequencies were rather low and in a substantial amount of the families even equal to zero. Apart from the sample considerations already mentioned above (i.e., sample composition and size), other explanations concern the length and strength of the lab task. The paradigm of the lab task is promising, as changes in skin conduct levels showed that a measurable amount of stress was induced and the proximity seeking reaction to the stress (i.e., how long the children waited to use the bell) was longitudinally related to the development of depression in the total sample [22], of which we studied a subset. Nevertheless, we cannot be sure that the level of distress and the bell manipulation were strong enough to elicit attachment-related behavior in all children. Equally, the task might have been too short to elicit all coded behaviors in a child. To this end, constructing a task that elicits attachment-related behavior in a different way would be advisable, as this would allow to investigate the replicability and generalizability of our findings.

Finally, critical evaluation of the MCAM coding scheme is warranted. On the positive side, almost all behaviors included in the scheme were found in our sample of 55 dyads (only controlling/contact-maintaining behavior was not observed). Moreover, there were clear differences between the dyads in how often the behaviors occurred. However, the relationship between the MCAM categories and the macro-coding scales from which they originate could profit from some further clarification. Indeed, certain behaviors that are rated in the macro-coding scales (e.g., warmth of the relationship), have no counterpart in the micro-coding system because they are not easily translated in singular behaviors or because they are difficult to detect in a short time interval. For future research, it would be interesting to use both macro-coding and the micro-coding on the same sample to investigate their unique and shared relations with attachment measures.

### Implications for theory

The findings line up with several theoretical models about attachment in middle childhood. First, we already highlighted that our findings resonate with the mutually responsive organization theory postulated by Kochanska [25]. Second, according to the supervisory partnership model of middle childhood attachment [17], securely attached children take more initiative and interact more actively with their parents. The findings of our study indeed show that less securely attached children were more reserved, took less initiative and even reacted less on specific parental behaviors. Third, the findings suggest, that Cassidy’s theory about anxiety and avoidance in infancy [2] also applies to observed attachment-related behavior in middle childhood. Finally, our study adds to the macro-coding, approaches of Brumariu et al. [5] and Boldt et al. [10], by demonstrating that the sequencing likelihood of micro-coded behaviors is linked to self-reported attachment, and by offering a short age adequate stress-inducing task and a rigorous analysis approach to obtain a clear picture of the dynamics.

### Practical implications

In this study, we related the relative frequencies and sequencing of attachment-related behaviors to self-reported attachment and found meaningful behavior patterns. These findings open new perspectives for interventions. First, we found that each dyad engages in the exchange of positive behavior, though the results need to be replicated in a more clinical sample. This provides a starting point for interventions that, like the circle of security [49] strengthen the positive interaction to make children more securely attached. Second, the circles of negativity in which low-trust children and their parents get stuck can be addressed via mother, child and by working on the interaction patterns. Minimizing negative and increasing positive child reactions to negative maternal behavior or vice versa, for example by training emotion regulation strategies or working on negative expectation biases [50,51], could lead to more satisfactory interactions. As a result both children and mothers could feel more in charge of the interaction and have more confidence in each other. Finally, we think that the coding paradigm could be a valuable research tool, which could be applied to clinical populations and inform therapeutic practice via research findings.

## Conclusion

This study proposed a micro-coding approach that allows to capture attachment-related interaction dynamics between mother and child in middle childhood, and investigate how they differ in function of attachment quality. Quantifying behavioral frequencies and sequences in a standardized stress-inducing lab task and depicting them in an easy-to-interpret network picture reveals that positive mother and child behaviors dynamically follow upon each other in all mother-child dyads. Relating the measures to self-reported attachment additionally shows that children with lower scores on trust and higher ones on avoidance tend to handle negative mother behavior less well. More anxiously attached children react positive on collaborative interactions.

## Supporting information

S1 Table Relative frequencies of mother and child behaviors of the MCAM. Total time of occurrence, number of dyads showing the behavior (Dyads), mean, standard deviation (SD) and range (Min, Max) for the main sample, and proportion of time a behavior is shown in the low trust dyad (Proportion) and z-scores (z-scores) comparing the low-trust dyad to the main sample. Full table.
(PDF)

S2 Table Sequences of mother and child behaviors. Number of dyads showing the sequence (Dyads), proportion (PropDy>0) of families showing the sequence more than would be expected by chance, mean and significance level (sig), standard deviation (SD) and range (Min, Max) for the main sample, and proportion of time a sequence is shown in the low trust dyad (sequence) and z-scores comparing the low-trust dyad to the main sample. Full table.
(PDF)

S3 Table Correlations between sequences of behavior and self-reported attachment. Spearman correlations for Trust, Avoidance and Anxiety and partial correlations for Avoidance and Anxiety (controlling pairwise for each other) between sequences of behavior and self-reported attachment (p-values between brackets).
(PDF)

S1 Fig Trust network. Node size (resp. thickness of the links) depict the absolute value of the spearman correlation between relative frequency (resp. sequencing likelihood) and the trust subscale of the PIML. Shading of links and node borders indicates positive (grey) or negative (black) correlations. Only significant links are depicted.
(PDF)

S2 Fig Avoidant network. (A) spearman correlations; (B) partial spearman correlations (correcting for anxiety). Node size (resp. thickness of the links) depicts absolute value of the correlation between relative frequency (resp. sequencing likelihood) and the avoidant subscale of the ECR-RC. Thicker borderline indicate significantly related behaviors, degree of significance is indicated with asterisk (**: p < .01). Shading or the node border and of the links indicates positive (grey) or negative (black) correlations. Only significant links are depicted.
(PDF)

S3 Fig Anxiety network. A) spearman correlations; B) partial spearman correlations (correcting for avoidance). Node size (resp. thickness of the links) depicts the absolute value of the correlation between the relative frequency (resp. sequencing likelihood) and the anxious subscale of the ECR-RC. Thicker node border indicate significantly related behaviors, the degree of significance is indicated with asterisk (**: p < .01). Shading of node border and links indicates positive (grey) or negative (black) correlations. Only significant links are depicted.
(PDF)

S4 Fig Visualization of the relative frequencies and sequencing likelihoods of the low-trust dyad. (A) Average main sample network, (B) network of the low-trust dyad, (C) z-scores of the low-trust dyad compared to the main sample. (A) & (B) Node size indicates the proportion of time a behavior is shown, the links are based on the values on 2-sec lagged data (one unit). Solid arrows depict sequences that are shown more than expected, dashed those that are shown less than expected. In (C) node size (resp. thickness of the links) indicates the absolute value of the z-scores. Behaviors with positive z-scores have a light gray border, while those of the negative ones is black. Considering sequences the positive z-scores are depicted as solid lines, while the negative ones are dashed. Please note that a minimum node size was introduced, to warranty the readability of the node labels. For the same reason, auto loops have been downscaled, to depict the between behavior sequences more clearly.
(PDF)

S1 Text Visualization of the correlations between self-reported attachment and relative frequencies and sequences in networks. (PDF)

S2 Text Visualization of the relative frequencies and sequencing likelihoods of the low-trust dyad. (PDF)
