# Supplementary material for: Unraveling middle childhood attachment-related behavior sequences using a micro-coding approach
Source: PLoS One. 2019 Oct 29;14(10):e0224372. doi: 10.1371/journal.pone.0224372 (PMC6818776; doi:10.1371/journal.pone.0224372)
Supplement: S1 Table — Total time of occurrence, number of dyads showing the behavior (Dyads), mean, standard deviation (SD) and range (Min, Max) for the main sample, and proportion of time a behavior is shown in the low trust dyad (Proportion) and z-scores (z-scores) comparing the low-trust dyad to the main sample. Full table. (PDF) [file pone.0224372.s001.pdf]

S1 Table. Relative frequencies of mother and child behaviors of the MCAM.

	Sample (*n*=54)						Low-trust dyad (*n*=1)	
	**Total time**	**Dyads**	**Mean**	**SD**	**Min**	**Max**	**Proportion**	***z*-scores**
**M+**	2077	54	.45	.14	.13	.83	.48	.17
**M-**	95	18	.02	.06	.00	.38	.13	2.04
**MAlone**	602	47	.13	.11	.00	.36	.41	2.49
**Together**	162	39	.04	.05	.00	.31	.01	-.49
**C+**	1783	54	.39	.17	.06	.78	.16	-1.39
**C-**	244	38	.05	.08	.00	.39	.60	7.21
**CAlone**	2179	53	.47	.22	.00	.92	.09	-1.76

Total time of occurrence, number of dyads showing the behavior (Dyads), mean, standard deviation (SD) and range (Min, Max) for the main sample, and proportion of time a behavior is shown in the low trust dyad (Proportion) and z-scores (z-scores) comparing the low-trust dyad to the main sample.
