# Supplementary material for: Unraveling middle childhood attachment-related behavior sequences using a micro-coding approach
Source: PLoS One. 2019 Oct 29;14(10):e0224372. doi: 10.1371/journal.pone.0224372 (PMC6818776; doi:10.1371/journal.pone.0224372)
Supplement: S2 Table — Number of dyads showing the sequence (Dyads), proportion (PropDy>0) of families showing the sequence more than would be expected by chance, mean and significance level (sig), standard deviation (SD) and range (Min, Max) for the main sample, and proportion of time a sequence is shown in the low trust dyad (sequence) and z-scores comparing the low-trust dyad to the main sample. Full table. (PDF) [file pone.0224372.s002.pdf]

S2 Table. Sequences of mother and child behaviors.

	Population (*n*=54)							Outlier (*n*=1)	
	Dyads	PropDy>0	Mean	sig	SD	Min	Max	sequence	z-scores
M+ **=>** M+	54	.94	.19	***	.11	-.05	.53	.32	1.14
M- **=>** M+	18	.56	.00		.04	-.25	.08	-.09	-2.22
MAlone **=>** M+	47	.40	-.01		.07	-.16	.28	.02	.33
Together => M+	39	.46	.00		.03	-.06	.09	.01	.38
C+ => M+	54	.78	.07	***	.10	-.17	.29	.08	.13
C- => M+	37	.65	.01		.03	-.07	.14	-.04	-1.65
CAlone => M+	53	.19	-.06		.07	-.23	.08	-.07	-.14
M+ => M-	18	.39	-.01		.04	-.27	.05	-.11	-2.67
M- => M-	18	.61	.15	**	.18	-.02	.65	.69	3.06
MAlone => M-	15	.07	-.01		.01	-.04	.01	-.05	-4.88
Together => M-	11	.09	.01		.06	-.03	.39	-.01	-.28
C+ => M-	18	.56	.00		.03	-.23	.04	-.08	-2.23
C- => M-	15	.20	.01		.06	-.05	.31	.06	.92
CAlone => M-	17	.29	.00		.05	-.07	.33	-.06	-1.26
M+ => MAlone	47	.53	.00		.06	-.16	.22	-.01	-.19
M- => MAlone	15	.20	.00		.01	-.03	.07	.00	.05
MAlone => MAlone	47	.83	.27	***	.20	-.04	.81	.47	1.01
Together => MAlone	36	.39	.03		.15	-.07	1	.02	-.12

Number of dyads showing the sequence (Dyads), proportion (PropDy>0) of families showing the sequence more than would be expected by chance , mean and significance level (sig), standard deviation (SD) and range (Min, Max) for the main sample, and proportion of time a sequence is shown in the low trust dyad (sequence) and z-scores comparing the low-trust dyad to the main sample.

Note: an upper-tailed one sample t-test was used. * p<.05; ** p<.01, ***p<.001

	Population (*n*=54)							Outlier (*n*=1)	
	Dyads	PropDy>0	Mean	sig	SD	Min	Max	sequence	z-scores
C+ => MAlone	47	.15	-.04		.05	-.16	.12	-.01	.44
C- => MAlone	31	.16	.00		.05	-.11	.28	-.17	-3.50
CAlone => MAlone	46	.20	-.07		.08	-.26	.03	-.09	-.17
M+ => Together	39	.56	.00		.03	-.10	.05	.01	.51
M- => Together	11	.09	.00		.02	-.03	.12	-.01	-.54
MAlone => Together	36	.50	.04	*	.15	-.05	1	.02	-.18
Together => Together	39	.56	.17	***	.18	-.03	.66	-.01	-.96
C+ => Together	39	.28	-.01		.03	-.09	.09	.07	2.67
C- => Together	25	.20	.00		.03	-.07	.11	-.01	-.43
CAlone **=>** Together	39	.33	-.01		.04	-.13	.07	-.01	.12
M+ **=>** C+	54	.78	.08	***	.10	-.09	.31	.11	.36
M- **=>** C+	18	.22	.00		.03	-.16	.10	-.08	-2.47
MAlone **=>** C+	47	.30	-.02		.06	-.19	.15	.02	.65
Together **=>** C+	39	.38	-.01		.02	-.12	.06	.07	2.94
C+ **=>** C+	54	.98	.24	***	.12	.00	.51	.31	.59
C- **=>** C+	37	.16	-.02		.03	-.12	.05	-.10	-2.43
CAlone **=>** C+	53	.45	-.01		.10	-.27	.26	-.06	-.58
M+ **=>** C-	35	.51	.01	*	.06	-.05	.30	-.04	-1.02
M- **=>** C-	14	.50	.01		.04	-.06	.19	.09	1.82
MAlone **=>** C-	29	.21	.00		.04	-.14	.13	-.12	-3.04

Number of dyads showing the sequence (Dyads), proportion (PropDy>0) of families showing the sequence more than would be expected by chance , mean and significance level (sig), standard deviation (SD) and range (Min, Max) for the main sample, and proportion of time a sequence is shown in the low trust dyad (sequence) and z-scores comparing the low-trust dyad to the main sample.

Note: an upper-tailed one sample t-test was used. * p<.05; ** p<.01, ***p<.001

	Population (*n*=54)							Outlier (*n*=1)	
	Dyads	PropDy>0	Mean	sig	SD	Min	Max	sequence	z-scores
Together **=>** C-	23	.13	.00		.02	-.04	.10	-.01	-.46
C+ **=>** C-	35	.17	-.02		.04	-.13	.05	-.02	.06
C- **=>** C-	34	.59	.12	***	.15	-.03	.48	.31	1.29
CAlone **=>** C-	34	.53	.00		.03	-.08	.06	.00	.23
M+ **=>** CAlone	53	.28	-.05		.08	-.29	.14	-.04	.13
M- **=>** CAlone	17	.24	-.01		.02	-.07	.10	-.06	-2.35
MAlone **=>** CAlone	46	.15	-.07		.07	-.23	.01	-.09	-.19
Together **=>** CAlone	39	.26	-.02		.03	-.11	.05	-.01	.21
C+ **=>** CAlone	53	.45	.00		.09	-.25	.22	-.06	-.63
C- **=>** CAlone	36	.42	-.01		.03	-.13	.05	.02	1.05
CAlone **=>** CAlone	53	.98	.47	***	.18	-.02	.81	.58	.63

Number of dyads showing the sequence (Dyads), proportion (PropDy>0) of families showing the sequence more than would be expected by chance , mean and significance level (sig), standard deviation (SD) and range (Min, Max) for the main sample, and proportion of time a sequence is shown in the low trust dyad (sequence) and z-scores comparing the low-trust dyad to the main sample.

Note: an upper-tailed one sample t-test was used. * p<.05; ** p<.01, ***p<.001
