# Supplementary material for: Unraveling middle childhood attachment-related behavior sequences using a micro-coding approach
Source: PLoS One. 2019 Oct 29;14(10):e0224372. doi: 10.1371/journal.pone.0224372 (PMC6818776; doi:10.1371/journal.pone.0224372)
Supplement: S3 Table — Spearman correlations for Trust, Avoidance and Anxiety and partial correlations for Avoidance and Anxiety (controlling pairwise for each other) between sequences of behavior and self-reported attachment (p-values between brackets). (PDF) [file pone.0224372.s003.pdf]

S3 Table. Correlations between sequences of behavior and self-reported attachment.

	Trust	Avoidance	Avoidance (partial)	Anxiety	Anxiety (partial)
M+ => M+	-.36**(.01)	.36**(.01)	.42**(0)	.03(.86)	-.23(.09)
M- => M+	.14(.33)	-.03(.84)	-.03(.82)	0(.98)	.01(.92)
MAlone => M+	-.18(.19)	-.01(.95)	0(.99)	-.02(.9)	-.02(.91)
Together => M+	-.06(.65)	.02(.87)	-.06(.69)	.12(.38)	.13(.34)
C+ => M+	-.03(.84)	.14(.32)	.18(.2)	-.01(.92)	-.11(.43)
C- => M+	0(.98)	.19(.18)	.15(.28)	.11(.44)	0(.98)
CAlone => M+	-.11(.43)	-.04(.77)	-.16(.24)	.17(.23)	.23(.1)
M+ => M-	.3*(.03)	-.21(.12)	-.16(.24)	-.14(.31)	-.02(.86)
M- => M-	.01(.98)	.03(.91)	.12(.65)	-.07(.77)	-.14(.6)
MAlone => M-	.15(.29)	-.35*(.01)	-.25(.08)	-.27(.06)	-.1(.51)
Together => M-	-.07(.63)	-.28(.06)	-.21(.18)	-.2(.18)	-.04(.77)
C+ => M-	.23(.09)	-.13(.35)	-.14(.3)	-.02(.9)	.07(.64)
C- => M-	-.5**(0)	.14(.4)	.08(.62)	.12(.45)	.05(.74)
CAlone=> M-	.12(.4)	.09(.52)	.2(.14)	-.14(.32)	-.23(.1)
M+ => MAlone	-.12(.39)	-.05(.75)	-.08(.56)	.04(.78)	.08(.58)
M- => MAlone	-.08(.59)	-.15(.31)	-.12(.43)	-.09(.52)	-.01(.93)
MAlone => MAlone	-.37*(.01)	.19(.2)	.14(.34)	.12(.41)	.02(.91)
Together => MAlone	-.09(.54)	-.1(.49)	.02(.87)	-.21(.15)	-.18(.21)

Spearman correlations for Trust, Avoidance and Anxiety and partial correlations for Avoidance and Anxiety (controlling pairwise for each other) between sequences of behavior and self-reported attachment (p-values between brackets).

Note: significance levels: *: *p*<.05,** p<.01, *** p<.001. All correlations are spearman correlations. Partial correlations with anxiety control for avoidance and vice versa.

	Trust	Avoidance	Avoidance (partial)	Anxiety	Anxiety (partial)
C+ => MAlone	.12(.38)	-.09(.5)	-.01(.95)	-.15(.26)	-.12(.38)
C- => MAlone	-.02(.89)	-.19(.18)	-.07(.64)	-.24(.08)	-.17(.24)
CAlone => MAlone	.19(.16)	-.08(.56)	-.05(.7)	-.07(.63)	-.02(.86)
M+ => Together	-.19(.17)	.27*(.05)	.13(.34)	.3*(.03)	.18(.2)
M- => Together	-.07(.63)	-.28(.06)	-.21(.18)	-.2(.18)	-.04(.77)
MAlone => Together	-.06(.7)	-.07(.64)	-.06(.69)	-.04(.81)	0(.97)
Together =>Together	-.04(.83)	-.07(.68)	-.06(.73)	-.04(.81)	0(.99)
C+ => Together	-.01(.93)	-.07(.6)	-.09(.52)	.01(.97)	.06(.69)
C- => Together	.02(.86)	-.17(.23)	-.02(.9)	-.28*(.05)	-.23(.11)
CAlone => Together	-.02(.9)	.06(.7)	.02(.87)	.06(.65)	.04(.77)
M+ => C+	-.05(.72)	.24(.08)	.12(.4)	.26(.06)	.15(.28)
M- => C+	.23(.1)	-.36**(.01)	-.31*(.02)	-.19(.17)	.01(.92)
MAlone => C+	.13(.33)	.02(.88)	-.03(.85)	.08(.58)	.08(.58)
Together => C+	-.15(.27)	.12(.39)	-.07(.63)	.31*(.02)	.29*(.03)
C+ => C+	.16(.24)	-.22(.11)	-.2(.16)	-.11(.44)	.02(.88)
C- => C+	.16(.24)	-.2(.14)	-.16(.26)	-.13(.34)	-.02(.88)
CAlone => C+	-.07(.64)	-.12(.38)	-.09(.54)	-.09(.5)	-.03(.84)
M+ => C-	.18(.19)	.2(.15)	.22(.12)	.04(.77)	-.09(.53)
M- => C-	-.36*(.03)	.32*(.05)	.15(.36)	.37*(.02)	.24(.15)
MAlone => C-	.04(.8)	-.05(.72)	.1(.5)	-.23(.1)	-.24(.08)

Spearman correlations for Trust, Avoidance and Anxiety and partial correlations for Avoidance and Anxiety (controlling pairwise for each other) between sequences of behavior and self-reported attachment (p-values between brackets).

Note: significance levels: *: $p<.05$,** p<.01, *** p<.001. All correlations are spearman correlations. Partial correlations with anxiety control for avoidance and vice versa.

	Trust	Avoidance	Avoidance (partial)	Anxiety	Anxiety (partial)
Together => C-	-.01(.92)	-.22(.12)	-.03(.85)	-.38**(.01)	-.31*(.03)
C+ => C-	.14(.32)	-.26(.06)	-.17(.22)	-.22(.1)	-.1(.5)
C- => C-	-.39*(.01)	.2(.23)	.01(.97)	.35*(.03)	.3(.08)
CAlone => C-	-.1(.49)	.09(.53)	-.04(.78)	.21(.12)	.2(.15)
M+ => CAlone	-.04(.75)	.02(.91)	.14(.32)	-.17(.21)	-.22(.11)
M- => CAlone	-.12(.38)	.19(.17)	.22(.12)	.02(.9)	-.11(.44)
MAlone => CAlone	.17(.21)	-.11(.41)	-.07(.63)	-.11(.45)	-.05(.72)
Together => CAlone	-.14(.31)	.04(.8)	.03(.86)	.02(.86)	.01(.96)
C+ => CAlone	.07(.61)	-.04(.78)	-.09(.51)	.06(.64)	.1(.45)
C- => CAlone	-.07(.64)	.09(.5)	-.02(.88)	.2(.15)	.18(.2)
CAlone => CAlone	-.07(.64)	-.01(.97)	-.01(.96)	0(.99)	.01(.97)

Spearman correlations for Trust, Avoidance and Anxiety and partial correlations for Avoidance and Anxiety (controlling pairwise for each other) between sequences of behavior and self-reported attachment (p-values between brackets).

Note: significance levels: *: $p<.05$,** $p<.01$, *** $p<.001$. All correlations are spearman correlations. Partial correlations with anxiety control for avoidance and vice versa.
