# Supplementary figures and images for: Unraveling middle childhood attachment-related behavior sequences using a micro-coding approach
Source: PLoS One. 2019 Oct 29;14(10):e0224372. doi: 10.1371/journal.pone.0224372 (PMC6818776; doi:10.1371/journal.pone.0224372)

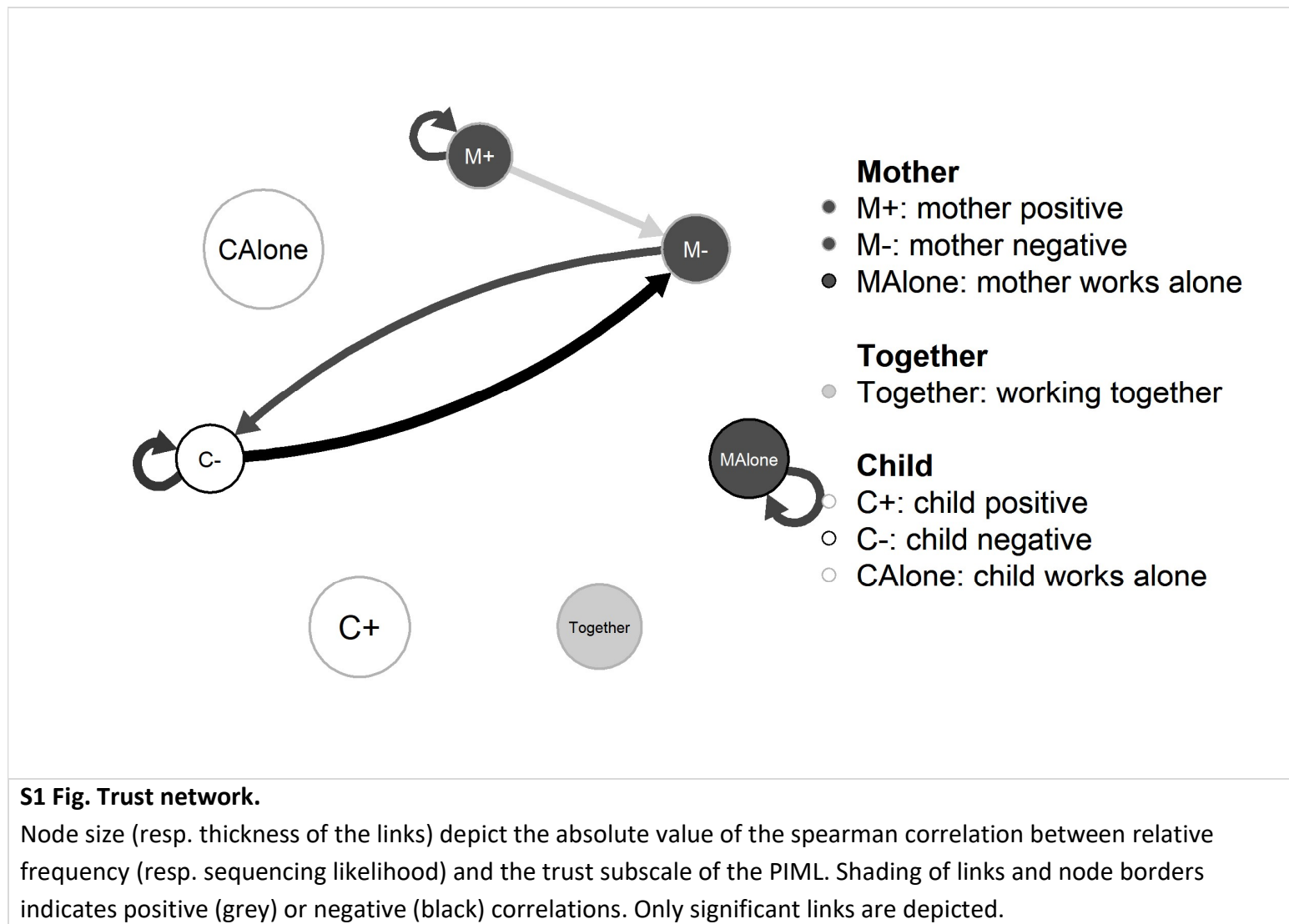

Supplement: S1 Fig — Node size (resp. thickness of the links) depict the absolute value of the spearman correlation between relative frequency (resp. sequencing likelihood) and the trust subscale of the PIML. Shading of links and node borders indicates positive (grey) or negative (black) correlations. Only significant links are depicted. (PDF) [file pone.0224372.s004.pdf]
