# Supplementary material for: Unraveling middle childhood attachment-related behavior sequences using a micro-coding approach
Source: PLoS One. 2019 Oct 29;14(10):e0224372. doi: 10.1371/journal.pone.0224372 (PMC6818776; doi:10.1371/journal.pone.0224372)
Supplement: S2 Fig — (A) spearman correlations; (B) partial spearman correlations (correcting for anxiety). Node size (resp. thickness of the links) depicts absolute value of the correlation between relative frequency (resp. sequencing likelihood) and the avoidant subscale of the ECR-RC. Thicker borderline indicate significantly related behaviors, degree of significance is indicated with asterisk (**: p < .01). Shading or the node border and of the links indicates positive (grey) or negative (black) correlations. Only significant links are depicted. (PDF) [file pone.0224372.s005.pdf]

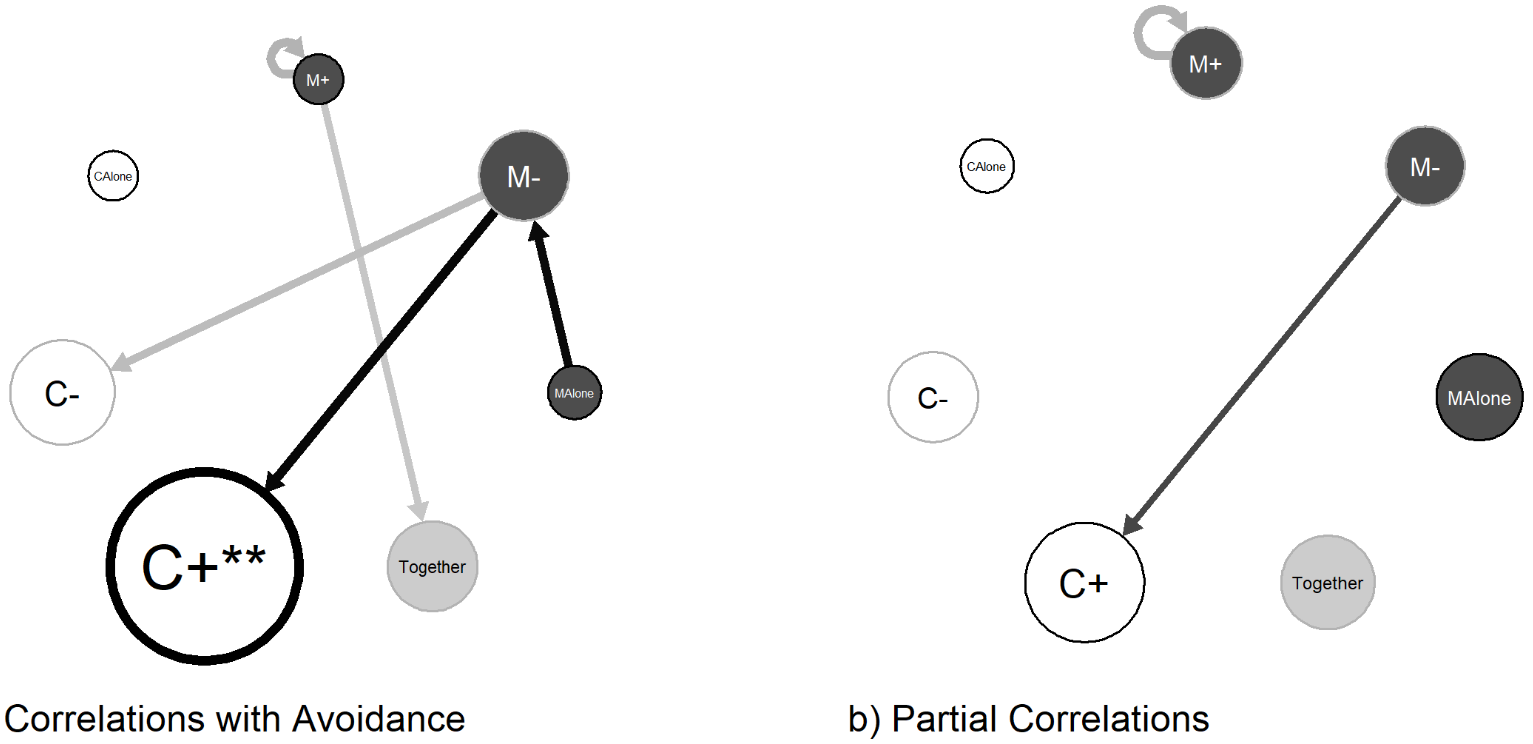

S2 Fig. Avoidant network.

a) spearman correlations; b) partial spearman correlations (correcting for anxiety). Node size (resp. thickness of the links) depicts absolute value of the correlation between relative frequency (resp. sequencing likelihood) and the avoidant subscale of the ECR-RC. Thicker borderline indicate significantly related behaviors, degree of significance is indicated with asterisk (**: p<.01). Shading or the node border and of the links indicates positive (grey) or negative (black) correlations. Only significant links are depicted.
