# Supplementary material for: Unraveling middle childhood attachment-related behavior sequences using a micro-coding approach
Source: PLoS One. 2019 Oct 29;14(10):e0224372. doi: 10.1371/journal.pone.0224372 (PMC6818776; doi:10.1371/journal.pone.0224372)
Supplement: S3 Fig — A) spearman correlations; B) partial spearman correlations (correcting for avoidance). Node size (resp. thickness of the links) depicts the absolute value of the correlation between the relative frequency (resp. sequencing likelihood) and the anxious subscale of the ECR-RC. Thicker node border indicate significantly related behaviors, the degree of significance is indicated with asterisk (**: p < .01). Shading of node border and links indicates positive (grey) or negative (black) correlations. Only significant links are depicted. (PDF) [file pone.0224372.s006.pdf]

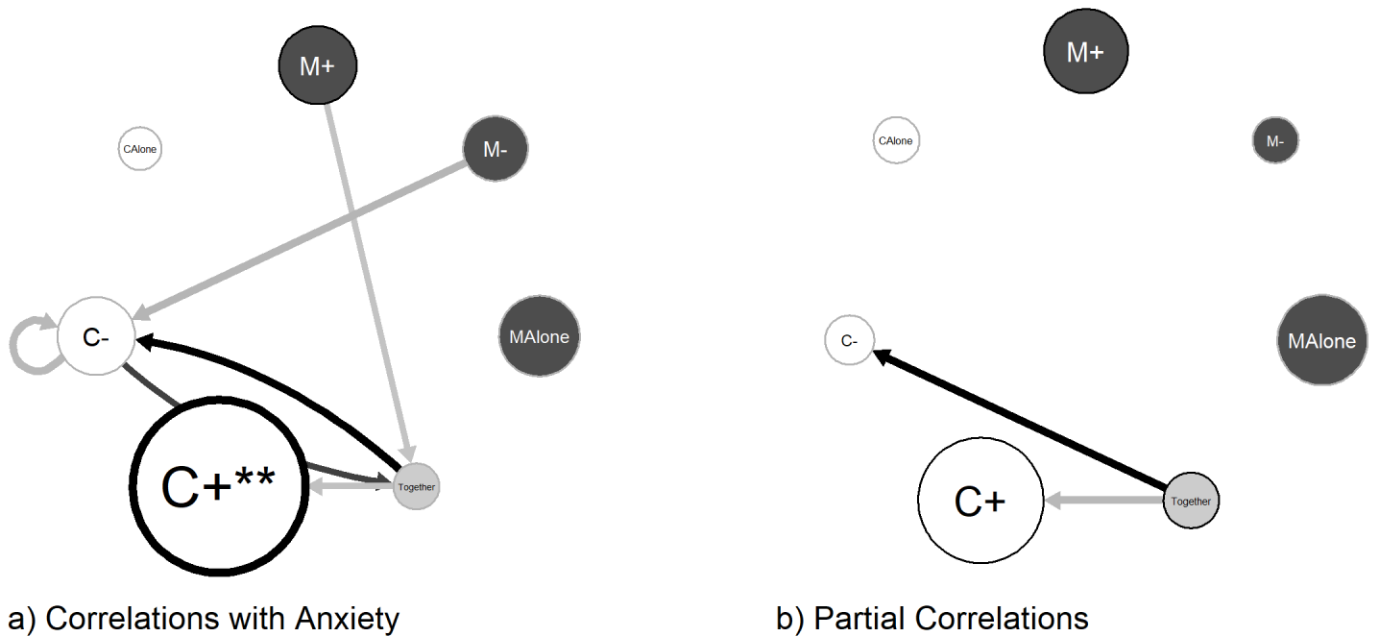

S3 Fig. Anxiety network.

a) spearman correlations; b) partial spearman correlations (correcting for avoidance). Node size (resp. thickness of the links) depicts the absolute value of the correlation between the relative frequency (resp. sequencing likelihood) and the anxious subscale of the ECR-RC. Thicker node border indicate significantly related behaviors, the degree of significance is indicated with asterisk (**: p<.01). Shading of node border and links indicates positive (grey) or negative (black) correlations. Only significant links are depicted.
