# Supplementary material for: Unraveling middle childhood attachment-related behavior sequences using a micro-coding approach
Source: PLoS One. 2019 Oct 29;14(10):e0224372. doi: 10.1371/journal.pone.0224372 (PMC6818776; doi:10.1371/journal.pone.0224372)
Supplement: S4 Fig — (A) Average main sample network, (B) network of the low-trust dyad, (C) z-scores of the low-trust dyad compared to the main sample. (A) & (B) Node size indicates the proportion of time a behavior is shown, the links are based on the values on 2-sec lagged data (one unit). Solid arrows depict sequences that are shown more than expected, dashed those that are shown less than expected. In (C) node size (resp. thickness of the links) indicates the absolute value of the z-scores. Behaviors with positive z-scores have a light gray border, while those of the negative ones is black. Considering sequences the positive z-scores are depicted as solid lines, while the negative ones are dashed. Please note that a minimum node size was introduced, to warranty the readability of the node labels. For the same reason, auto loops have been downscaled, to depict the between behavior sequences more clearly. (PDF) [file pone.0224372.s007.pdf]

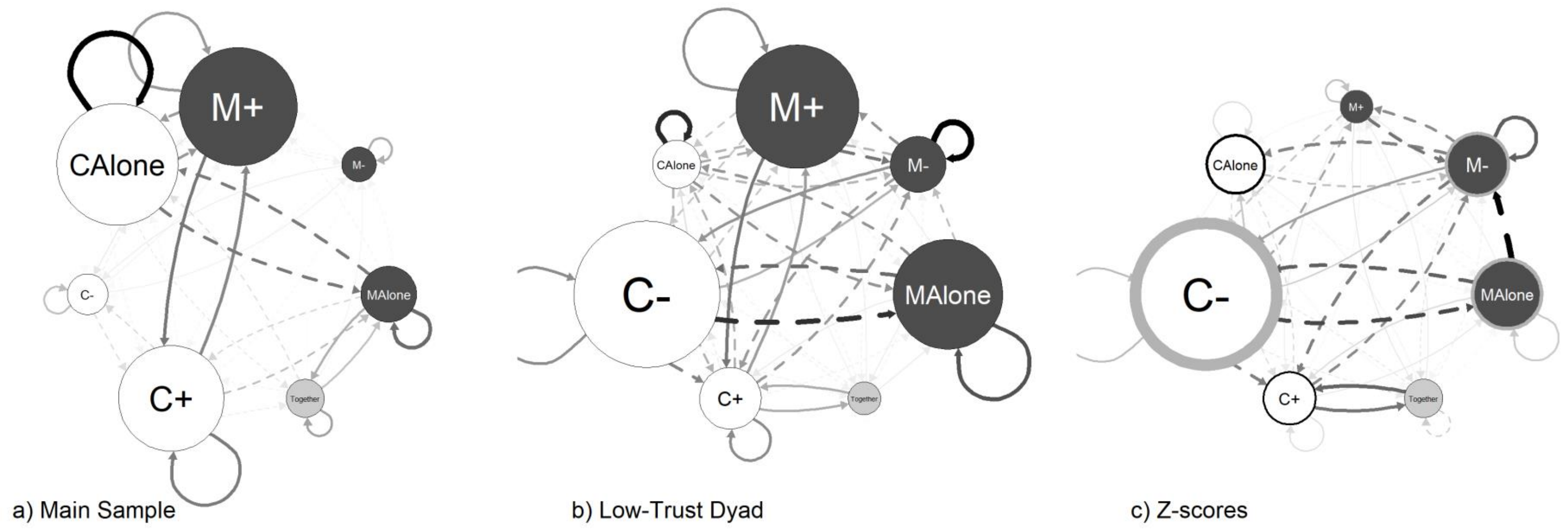

S4 Fig. Visualization of the relative frequencies and sequencing likelihoods of the low-trust dyad.
(a) Average main sample network, (b) network of the low-trust dyad, (c) *z*-scores of the low-trust dyad compared to the main sample. (a)&(b) Node size indicates the proportion of time a behavior is shown, the links are based on the values on 2-sec lagged data (one unit). Solid arrows depict sequences that are shown more than expected, dashed those that are shown less than expected. In (c) node size (resp. thickness of the links) indicates the absolute value of the z-scores. Behaviors with positive *z*-scores have a light gray border, while those of the negative ones is black. Considering sequences the positive *z*-scores are depicted as solid lines, while the negative ones are dashed. Please note that a minimum node size was introduced, to warranty the readability of the node labels. For the same reason, auto loops have been downscaled, to depict the between behavior sequences more clearly.
