# Supplementary material for: Unraveling middle childhood attachment-related behavior sequences using a micro-coding approach
Source: PLoS One. 2019 Oct 29;14(10):e0224372. doi: 10.1371/journal.pone.0224372 (PMC6818776; doi:10.1371/journal.pone.0224372)
Supplement: S1 Text — (PDF) [file pone.0224372.s008.pdf]

S1 Text. Visualization of the correlations in networks.

The resulting correlations are visualized in three separate networks (Figs S1 to S3). In these networks, the node size depicts the absolute value of the correlation between the corresponding relative frequency and attachment measure. Significant correlations are indicated by an asterisk and a thickened node border. The shading of the border indicates the direction of the relationship, with black borders reflecting a negative correlation, and grey borders a positive one. The directed links between the nodes indicate that the corresponding sequencing likelihood correlates significantly with a self-report measure, with the link's thickness depicting the size of the correlation and the shading the direction (black=negative correlation, grey=positive correlation).
