# Supplementary material for: Unraveling middle childhood attachment-related behavior sequences using a micro-coding approach
Source: PLoS One. 2019 Oct 29;14(10):e0224372. doi: 10.1371/journal.pone.0224372 (PMC6818776; doi:10.1371/journal.pone.0224372)
Supplement: S2 Text — (PDF) [file pone.0224372.s009.pdf]

S2 Text. Visualization of the relative frequencies and sequencing likelihoods of the low-trust dyad.

Fig S4 shows that three behavior categories occurred considerably more often in the low-trust dyad when compared to the average of the main sample: C-, M-, and MAlone. Regarding sequencing likelihood, the M+→C+ and C+→ M+ sequences were still more likely than expected by chance and did not differ markedly from the main sample average. These links were, however, complemented with additional negative links (e.g., M- →C-) of which the likelihood clearly exceeded the sample average, while other links were less likely (e.g., M- →C+).
